# Aqueous-based tissue clearing in crustaceans

**DOI:** 10.1186/s40851-018-0099-6

**Published:** 2018-06-06

**Authors:** Alu Konno, Shigetoshi Okazaki

**Affiliations:** Department of Medical Spectroscopy, Hamamatsu University School of Medicine, 1-20-1 Handayama, Higashi-ku, Hamamatsu-City, Shizuoka-Pref 431-3192 Japan

**Keywords:** Tissue clearing, Advanced CUBIC, Crustacea, Isopoda, Decapoda

## Abstract

**Background:**

Investigation of the internal tissues and organs of a macroscopic organism usually requires destructive processes, such as dissection or sectioning. These processes are inevitably associated with the loss of some spatial information. Recently, aqueous-based tissue clearing techniques, which allow whole-organ or even whole-body clearing of small rodents, have been developed and opened a new method of three-dimensional histology. It is expected that these techniques will be useful tools in the field of zoology, in which organisms with highly diverse morphology are investigated and compared. However, most of these new methods are optimized for soft, non-pigmented organs in small rodents, especially the brain, and their applicability to non-model organisms with hard exoskeletons and stronger pigmentation has not been tested.

**Results:**

We explored the possible application of an aqueous-based tissue clearing technique, advanced CUBIC, on small crustaceans. The original CUBIC procedure did not clear the terrestrial isopod, *Armadillidium vulgare*. Therefore, to apply the whole-mount clearing method to isopods with strong pigmentation and calcified exoskeletons, we introduced several pretreatment steps, including decalcification and bleaching. Thereafter, the clearing capacity of the procedure was dramatically improved, and *A. vulgare* became transparent. The internal organs, such as the digestive tract and male reproductive organs, were visible through sclerites using an ordinary stereomicroscope. We also found that fluorescent nuclear staining using propidium iodide (PI) helped to visualize the internal organs of cleared specimens. Our procedure was also effective on the marine crab, *Philyra* sp.

**Conclusions:**

In this study, we developed a method to clear whole tissues of crustaceans. To the best of our knowledge, this is the first report of whole-mount clearing applied to crustaceans using an aqueous-based technique. This technique could facilitate morphological studies of crustaceans and other organisms with calcified exoskeletons and pigmentation.

**Electronic supplementary material:**

The online version of this article (10.1186/s40851-018-0099-6) contains supplementary material, which is available to authorized users.

## Background

Biological structures are three-dimensional (3D). It is generally difficult to observe the 3D structures and spatial relationships of internal organs in opaque organisms. Traditionally, this limitation was overcome using 3D reconstruction from serial sections [1, 2]. However, serial sectioning is usually painstaking, time-consuming, and limited to small specimens. Advanced imaging technologies, such as magnetic resonance imaging [3] and computed tomography [4], are powerful tools for imaging internal structures; however, these instruments have limited resolution compared to light microscopy, and are much less accessible to most zoologists.

Another strategy to observe internal structures is to make opaque organisms transparent. Although the concept of tissue clearing is over 100-years-old [5], its use has been relatively limited to the field of osteology. Recently, advances in genetically encoded fluorescent markers and the advent of various optical sectioning microscopies have stimulated the development of new aqueous-based tissue clearing techniques [6, 7]. We considered that these novel techniques have the potential to reform current experimental designs and advance our understanding on the morphology of a wide range of organisms. However, most of the new tissue clearing techniques are designed and optimized for the soft tissues of small rodents, and their applicability to hard tissues or other organisms has scarcely been explored. A recent study reported that mouse bony tissues could be cleared using an aqueous-based method coupled with the decalcification and decoloration of heme [8]. Here, we tested the possible application of aqueous-based tissue clearing on crustaceans. Since crustaceans have a hard exoskeleton and strong pigmentation, which hamper the observation of internal structures, successful application of tissue clearing techniques would facilitate morphological and histological studies of this taxon.

In this study, we attempted whole-body tissue clearing of small crustaceans using an aqueous-based technique, advanced CUBIC [9]. We found that the original protocol did not clear the terrestrial isopod, *Armadillidium vulgare*. Therefore, we introduced some pretreatment steps, including decalcification and bleaching. After optimizing the pretreatment, clearing efficiency was dramatically improved and most of the body parts became transparent. The same procedure was also effective for the marine crab, *Philyra* sp. The internal anatomy of cleared specimens was easily observed using stereomicroscopy. Further characteristics of some of the cellular arrangements were revealed using fluorescent nuclear staining. Our approach provides a useful tool for the morphological study of crustaceans, and possibly other animals with calcified body parts and/or pigmentation.

## Methods

### Reagents

All reagents were purchased from Wako Pure Chemical Industries (Osaka, Japan), except for the following: ethylenediaminetetraacetic acid (EDTA) (Dojindo Laboratories, Kumamoto, Japan), N,N,N′,N′-tetrakis(2-hydroxypropyl)ethylenediamine (Quadrol) (Tokyo Chemical Industry, Tokyo, Japan), Triton X-100 (Sigma-Aldrich, St. Louis, MO, USA), and propidium iodide (PI) (Thermo Fisher Scientific, Waltham, MA, USA).

### Animals

Common pill bugs, *A. vulgare*, were collected in Hamamatsu City, Japan. They were starved for about 24 h to empty the gut and were then fixed in 4% paraformaldehyde (PFA)/0.1 M phosphate buffer (PB) (pH 7.4). As immersion in the fixative causes them to roll up into a ball, we sandwiched them between stainless steel meshes in stretched form, and fixed them at 4 °C for at least 48 h. Marine crabs, *Philyra* sp., were collected in Shimoda Bay, and were fixed immediately in 10% formalin/sea water. They were kept in the fixative at room temperature (RT) until use. A hornet, *Vespa analis*, was collected in Hamamatsu City, Japan, and stored as a dry specimen. Before use, the hornet was rehydrated in PBS, and fixed in 4% PFA/0.1 M PB (pH 7.4) at 4 °C for 24 h.

### Pretreatment of samples for clearing

Samples were gently agitated on a shaker during all washing and incubation steps. The fixed animals were rinsed several times in PBS, and decalcified in 0.2 M EDTA (pH 8.0) at 4 °C for 24–48 h, with one change of the EDTA solution. Decalcified specimens were washed in PBS at RT. To minimize deformation during the procedures, samples were fixed again in 4% PFA/0.2 M PB (pH 7.4) overnight at 4 °C. Decalcified specimens were then bleached in hydrogen peroxide (H_2_O_2_)/PBS. To avoid vigorous reaction with H_2_O_2_, *A. vulgare* samples were first incubated in 0.03% H_2_O_2_/PBS at 37 °C until the formation of fine bubbles stopped (~ 24 h, but with significant variation among individuals). Since *Philyra* sp. did not bubble vigorously, this step was skipped. Then, *A. vulgare* and *Philyra* sp. samples were bleached in 3% H_2_O_2_/PBS for 12–48 h at 37 °C. The containers were not tightly closed to allow for the release of bubbles. The samples were then washed several times in PBS. When bubbles formed inside the gut, samples were transferred to an airtight container filled with degassed PBS at RT. Then it was capped without introducing air and kept at 4 °C to dissolve the bubbles.

### Whole-mount clearing

Whole-mount clearing was performed with the advanced CUBIC protocol [9]. Briefly, delipidation and refractive index (RI) matching were conducted with reagent-1 [25% (*w*/w) urea, 25% Quadrol, 15% (w/w) Triton X-100 in distilled water] and reagent-2 [25% (w/w) urea, 50% (w/w) sucrose, 10% (w/w) 2,2′,2″-nitrilotriethanol (triethanolamine) in distilled water], respectively. Decalcified and bleached samples were incubated in 1/2 reagent-1 (reagent-1:H_2_O = 1:1) for 6 h to overnight and then in 1× reagent-1 at 37 °C until they became transparent. The samples were washed several times in PBS and treated with 1/2 reagent-2 (reagent-2:PBS = 1:1) for more than 3 h. Then, samples were transferred to 1× reagent-2 and incubated until the solution became homogeneous. All steps were performed on a shaker at RT, except for the incubation in 1/2 and 1× reagent-1 at 37 °C.

Nuclear staining with PI was performed after the reagent-1 treatment. Following several PBS washes, samples were incubated in PBS containing 20 μg/ml PI overnight at 4 °C. After PI staining, samples were treated with reagent-2, as described above.

### Observations

Macroscopic images were photographed with a digital camera (Optio WG-2, Pentax, Tokyo, Japan). Stereomicroscopic images were obtained with an INFINITY*HD* camera (Luminera Corporation, Ontario, Canada) under oblique illumination. Fluorescence images of PI were obtained with WRAYCAM-SR130M camera (Wraymer, Osaka, Japan) with a filter for RFP. Both were mounted on an SZX16 stereomicroscope (Olympus, Tokyo, Japan).

## Results

### Optimization of pretreatments for whole-mount clearing

**Fig. 1:**
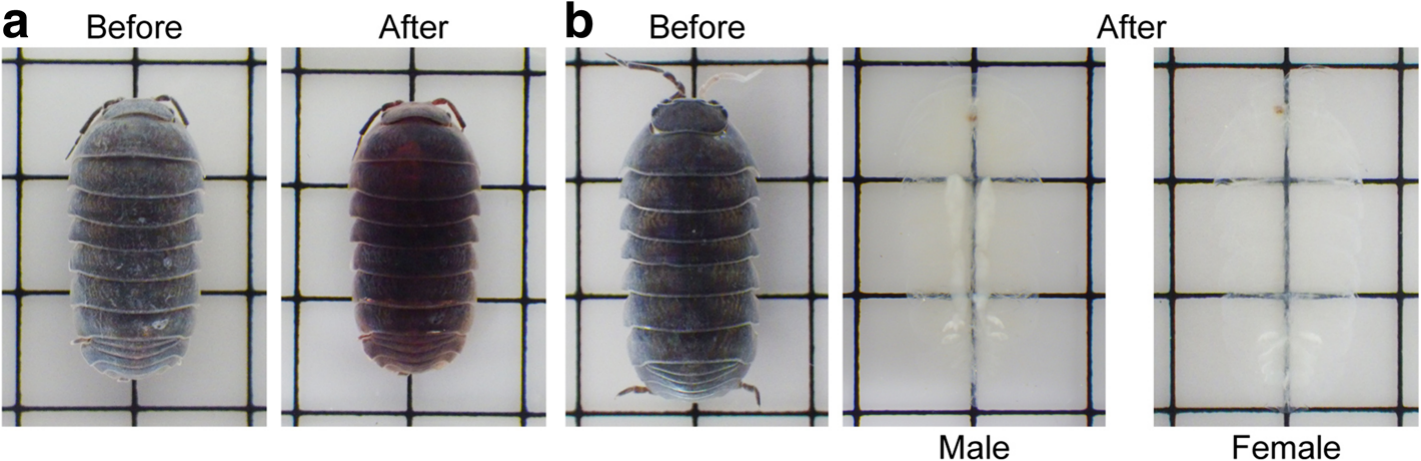
Whole-mount clearing of the terrestrial isopod, *A. vulgare*, with advanced CUBIC protocol. **a** *A. vulgare* cleared with advanced CUBIC protocol without any pretreatment. **b** Clearing after pretreatment, including decalcification and bleaching. Grid = 5 mm

We first assessed the applicability of the aqueous-based tissue clearing technique to crustaceans using the common pill bug, *A. vulgare*. We tested the advanced CUBIC [9] method due to its high tissue clearing capacity and the simplicity of the procedure [10, 11]. This method consists of two steps: (1) delipidation, decoloration, and hyperhydration in reagent-1 solution, followed by (2) refractive index (RI) matching in reagent-2 solution. Despite its powerful clearing capacity for various rodent tissues, this technique only rendered slight color change and no transparency in *A. vulgare* (Fig. 1a).

Most of the recently developed techniques have only been tested on tissues that lack hard components or pigments, except for heme and its derivatives. Therefore, we reasoned that the calcified exoskeleton and body pigmentation were barriers to effective tissue clearing in the isopod and introduced the decalcification and bleaching steps. After decalcification with EDTA, the solution turned slightly brownish, and the isopods were softened and changed in color. The same treatment had no effect on the hornet cuticle, which lacks calcium deposits (Additional file 1: Figure S1). Then *A. vulgare* samples were fixed again and bleached in H_2_O_2_ solution. Some individuals unexpectedly showed explosive bubbling upon contact with 3% H_2_O_2_ and their bodies were frequently broken apart. We resolved this problem by treating the samples with dilute (0.03%) H_2_O_2_ first. After the formation of fine bubbles stopped, they were safely transferred to a higher concentration of H_2_O_2_ solution for bleaching. Another problem we encountered was the formation of bubbles inside the gut of some individuals. This appeared not to damage tissues in the samples pretreated with dilute H_2_O_2_, but hampered microscopy after clearing. Degassing the samples using a vacuum pump was not effective, so samples were transferred into degassed PBS at RT and then the temperature was lowered to 4 °C to further increase the solubility of gas. After this treatment, bubbles were completely dissolved within one day.

**Fig. 2:**
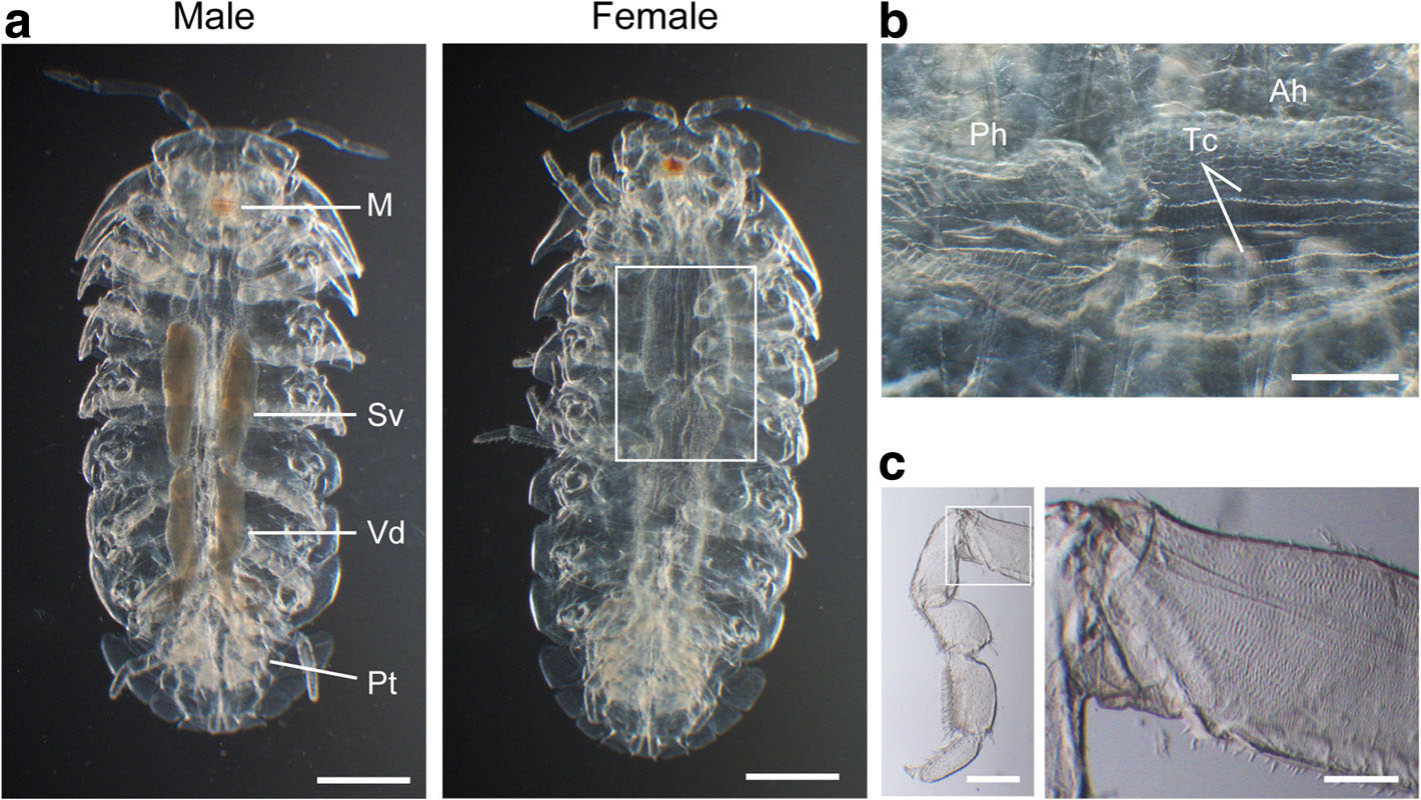
Stereoscopic examination of a cleared *A. vulgare*. **a** Dorsal view of a cleared male (left) and female (right). Most of the body parts became transparent, except for the sperm vesicle (Sv), vas deferens (Vd), mandibles (M), and pseudotrachea (Pt). Scale bars = 2 mm. The boxed region on the female is shown in **b** at higher magnification. **b** Junction of anterior (Ah) and posterior (Ph) hindgut. A pair of typhlosole channels (Tc) run along the dorsal midline of the anterior hindgut. Scale bar = 1 mm. **c** Muscle striation in a leg. The boxed region in the left panel is enlarged in the right panel. Scale bar = 500 μm (left), 200 μm (right). All observations were performed under appropriate oblique illumination

After optimizing the preclearing steps, the tissue clearing efficiency of the advanced CUBIC protocol was dramatically improved and most of the body parts became transparent (Fig. 1b). Males and females were distinguishable by the relatively low transparency of the testes and vas deferens [12] (Figs. 1 and 2). Stereomicroscopic observations confirmed good transparency of the cleared pill bugs, except for the jaw and respiratory structures in pleopods [13], as well as the male reproductive organs (Fig. 2a). The largest compartment of the digestive tract in pill bugs is the hindgut [14]. In cleared samples, the ordered lattice-like structure of the hindgut wall, a pair of typhlosole channels on the dorsal side of the anterior hindgut, and the junction between anterior and posterior hindguts were easily observed through the dorsal sclerites (Fig. 2b). At higher magnification, muscle striation in the legs was also observed (Fig. 2c).

These results indicate that the aqueous-based technique enabled whole-mount tissue clearing of small crustaceans after calcified deposits and pigments were removed.

### Visualization of internal structures with fluorescent nuclear staining

**Fig. 3:**
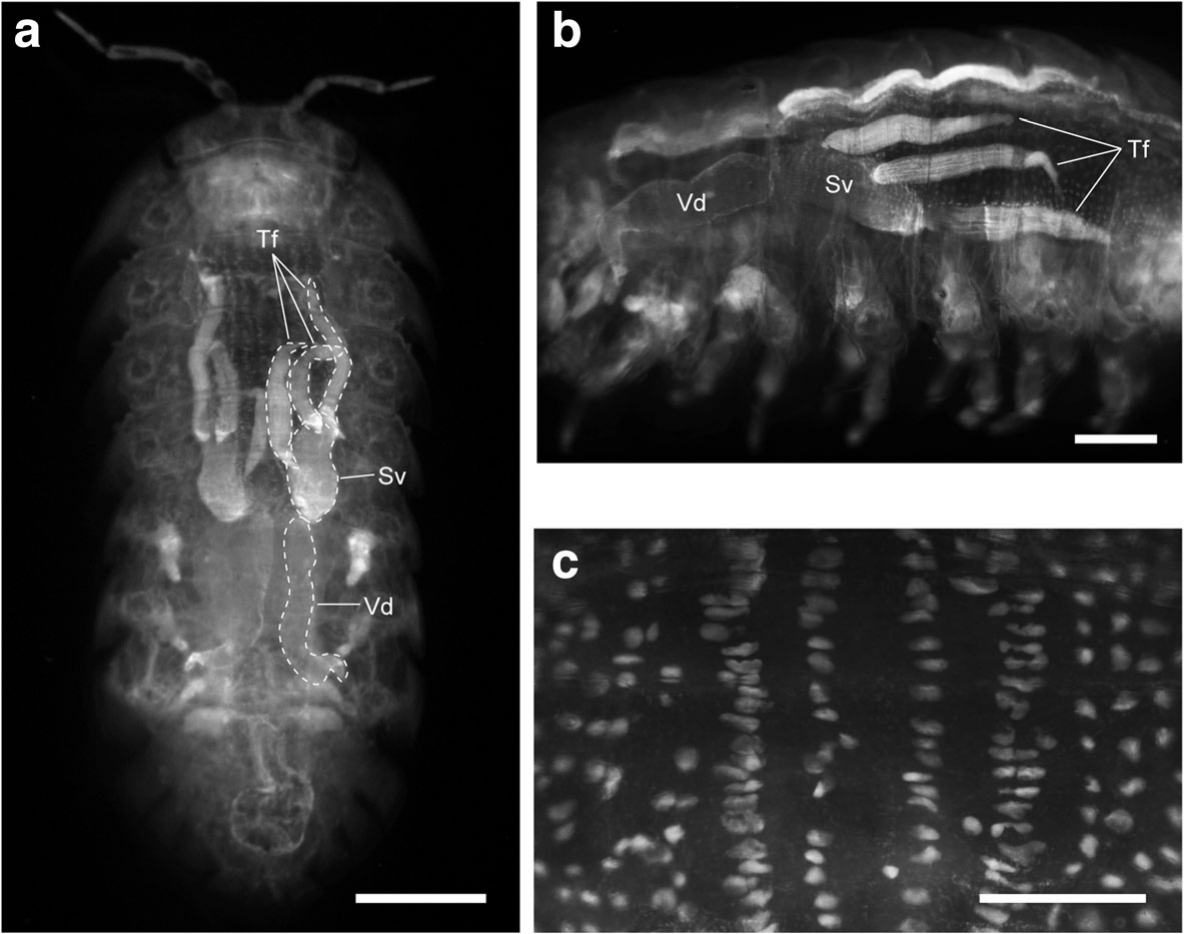
Propidium iodide (PI) staining of a cleared *A. vulgare* male. **a** Top view of the stained specimen. Dotted lines indicate the contour of male reproductive organs. Legs are removed. Scale bar = 2 mm. **b** Side view of the middle body part. Scale bar = 1 mm. Testis follicles (Tf), sperm vesicle (Sv), vas deferens (Vd). **c** Close-up image of the dorsal anterior hindgut. Large cell nuclei aligned in ordered rows. Scale bar = 500 μm

We used fluorescent nuclear staining to visualize anatomical structures in the cleared specimens (Fig. 3). PI staining revealed internal organs, especially the male reproductive system, of *A. vulgare*. The male reproductive system was similar to that of other terrestrial isopods [12]. Each of a pair of male reproductive organs is composed of three testis follicles, a seminal vesicle, and a vas deferens (Fig. 3a and b). Testis follicles [12], which were not visible in unstained samples (Fig. 2a), were clearly observed after PI staining (Fig. 3a and b). In the anterior hindgut, a characteristic array of cells was observed at a higher magnification. Large cell nuclei were arranged in ordered rows. The four dorsalmost rows, possible typhlosole channel cells, were particularly conspicuous by their large, laterally elongated nuclei (Fig. 3c).

These data suggest that the combination of whole-mount tissue clearing and fluorescent nuclear staining can reveal the arrangement and relationships of internal organs in non-model organisms.

### Whole-mount clearing of a small decapod

**Fig. 4:**
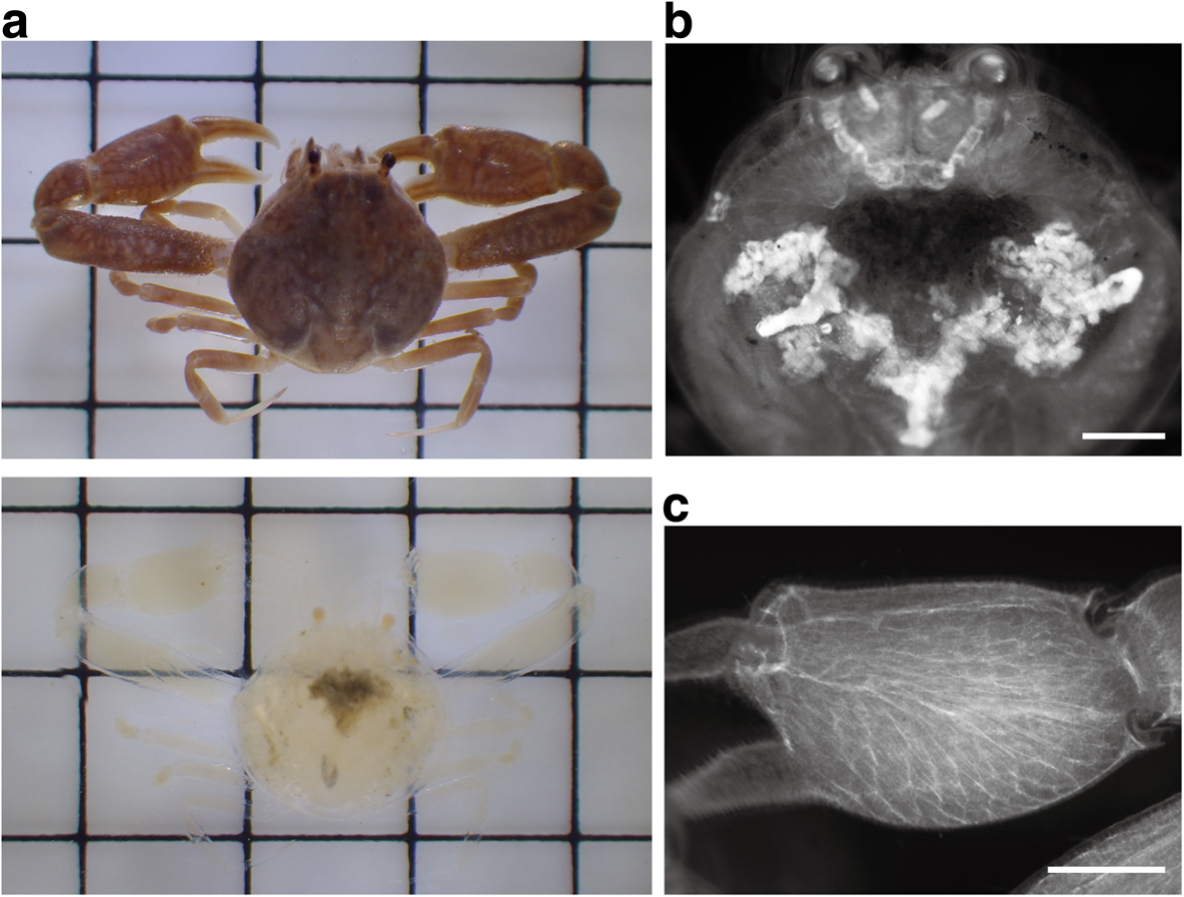
Whole-mount clearing of the marine crab, *Philyra* sp. **a** Specimen before (top) and after (bottom) clearing. Dark parts seen through the carapace are the gut content. Grid = 5 mm. **b**, **c** Nuclear staining with propidium iodide (PI). Dorsal view through the carapace and the right cheliped. Scale bars = 1 mm

Finally, we tested whether our procedure could be applied to another crustacean using the marine crab, *Philyra* sp. (Fig. 4). Its transparency was increased by decalcification alone (Additional file 1: Figure S1C). After bleaching and subsequent CUBIC procedure, this species was also successfully cleared. In this species, direct immersion in 3% H_2_O_2_ did not cause vigorous bubbling, and treatment with dilute H_2_O_2_ was omitted. Since they were not starved before fixation, the gut content was observed through the carapace (Fig. 4a). Fluorescent nuclear staining with PI revealed the hepatopancreas (Fig. 4b) and muscular architecture (Fig. 4c).

We conclude that tissue clearing with advanced CUBIC method after decalcification and bleaching is effective for various crustaceans.

## Discussion

In this study, we developed a whole-mount clearing method for use in crustaceans. To the best of our knowledge, this is the first report of an aqueous-based tissue clearing technique successfully applied to non-model invertebrates. Although a previous unique study has described a transparent composite prepared from the crab shell [15], this approach requires the complete removal of non-chitin components and is not suitable for histological applications. In comparison, the tissue clearing method described here could be subjected to various imaging analyses.

### Optimization of clearing steps

The main causes of tissue opacity are the presence of pigments and inhomogeneous RIs among cellular components and the medium [7]. Non-pigmented aquatic organisms are almost transparent when they have a similar RI to water [16]. Therefore, a general strategy for tissue clearing is the removal of pigments and the matching of RIs. For mammalian organs, the contribution of pigments to opacity is relatively small, except in some heme-rich organs. Conversely, strong pigmentation is a significant barrier to whole-mount tissue clearing of small invertebrates. In addition, calcified exoskeletons can hinder the effective penetration of clearing reagents into tissue components. Indeed, the advanced CUBIC method failed to clear *A. vulgare*. To resolve these problems, we introduced the pretreatments of decalcification with EDTA and bleaching with H_2_O_2_.

Decalcification has already been shown to be effective in clearing mammalian bony tissues [8, 17]. This step should help subsequent clearing processes by facilitating the penetration of clearing reagents. In addition, EDTA treatment alone improved the translucency of the calcified exoskeleton, especially in the crab (Additional file 1: Figure S1). This phenomenon is likely due to the reduced heterogeneity of RI caused by the removal of calcium deposits with high RI. This direct clearing effect of EDTA was not apparent in *A. vulgare*, probably because of its stronger pigmentation. EDTA solution turned slightly brownish during decalcification of the crustaceans, suggesting that some pigments, most likely the ones strongly associated with mineralized structures, are liberated. EDTA treatment did not have a visible effect on the exoskeleton of the hornet, suggesting that the improved transparency of crustaceans after EDTA treatment is purely caused by decalcification. In larger crustaceans, decalcified samples may become deformed after the loss of structural support. In this case, partial dissection may be required. Alternatively, hydrogel embedding methods, such as PACT [8, 17], may provide extra mechanical support.

We observed destructive bubbling in samples of *A. vulgare* during the bleaching step. Some *A. vulgare* bubbled vigorously upon contact with 3% H_2_O_2_. We overcame the problem by immersing samples in 0.03% H_2_O_2_ first. We also introduced a second fixation step after decalcification, based on the expectation that the removal of calcium deposits would unmask reactive groups that were not accessible during the first fixation. Although the cause of the different intensities of bubbling was unclear, variation in peroxidase activity during the molting cycle might be responsible. Indeed, several studies have reported the involvement and cyclical expression of peroxidases during ecdysozoan cuticular biosynthesis [18, 19]. Since the bubbling of *Philyra* sp. was much gentler, post-fixation and incubation with dilute H_2_O_2_ was not necessary. Therefore, this process would be simplified, depending on the species and lifecycle stage.

After bleaching, we encountered the problem of removing the bubbles formed in the lumen of the digestive tract. The bubbles do not hinder the latter clearing steps but can disturb the observation of cleared specimens. Degassing using a vacuum pump did not remove the bubbles and even damaged tissues. We found that the immersion of samples in a degassed buffer-filled container and storage at a lower temperature removed the bubbles. When no pump is available, immersion of specimens in a warm buffer and lowering the temperature would also be effective.

Tissue clearing with advanced CUBIC protocol became very effective after the pretreatments. In the cleared pill bugs, various organs were observed in situ. Some structures, such as part of the male reproductive system and pseudotrachea, were not effectively cleared. Although the reason for this was unclear, the RI of the immersion medium might not be sufficiently high for these structures. An immediate understanding of 3D anatomical structures in cleared whole-mount samples is one of the powerful and unique advantages of this procedure. Currently, tissue clearing techniques are used in combination with fluorescent reporter proteins and advanced microscopies. However, our results illustrate that whole-mount clearing of small animals can provide plenty of information, even when using a basic stereomicroscope.

### Staining of cleared samples

Although the internal anatomy of cleared *A. vulgare* could be observed without staining, specific staining methods made the technique more versatile. In zoology, it is often difficult to make use of genetically encoded marker proteins or good commercial antibodies. Therefore, the exploration of chemical probes, which is compatible with tissue clearing, is important. Small chemical probes also have an advantage of fast penetration into large specimens. Fluorescent nuclear staining is a popular technique used in aqueous-based tissue clearing to visualize the architecture of tissues and organs [9, 20]. We confirmed that nuclear staining with PI was useful and sometimes essential to observe the internal structures of cleared whole-mount crustaceans (Figs. 3 and 4). The non-biased visualization of cellular organization in whole-mount specimens using this type of staining might also facilitate the discovery of overlooked morphological characteristics.

Various staining methods are applied to samples cleared with aqueous-based procedures. For example, successful in situ hybridization was reported using CLARITY [21]. Some detergent-free clearing protocols, including SeeDB [22], FRUIT [23], and one of the Sca*l*eS variants [24], are compatible with lipophilic dyes [25, 26]. Very recently, Golgi-Cox staining for cleared brain samples was reported [27]. Although generalized protocols are not yet available for most of these techniques, it is worth testing their application in non-model organisms in future studies.

### Selection of tissue clearing techniques for zoologists

For researchers planning their first tissue clearing experiment, it is not easy to choose a suitable method from the many currently published clearing techniques [6, 7]. There is no gold standard, as every method has its own advantages and disadvantages. For zoologists, the first step is to test whether the organism of interest can be cleared, irrespective of the extent, since virtually no non-model organisms have been cleared. We believe that the advanced CUBIC method [9] is a good choice for preliminary experiments with various organisms. First, chemicals used in the procedure are non-toxic and inexpensive, and most are general reagents found in many laboratories. Second, it is a relatively easy method requiring only sequential changes in solutions. Finally, clearing using this method is faster than most of the other aqueous-based methods. The superior clearing capacity has also been reported in several comparative studies [10, 11]. One of the disadvantages of the technique is the temporary expansion of tissues during incubation in reagent-1. Since the expansion was offset during the washing and RI matching steps, this was not a problem in our experiments. The extent of expansion might be reduced with a modified CUBIC procedure (Reagent-1A protocol. http://cubic.riken.jp), where small amounts of NaCl are added to reagent-1 at a final concentration of ≥25 mM. In general, samples undergo expansion during delipidation with a high concentration detergent. Sca*l*eS [24], SeeDB [22], and FRUIT [23], which are reported to have little effect on sample size, might be suitable when tissue expansion is not acceptable. For fragile specimens, hydrogel-embedding using PACT [28, 29] might be useful; however, this approach also causes tissue expansion. Recently, another CUBIC protocol, CUBIC-L/R was published [20]. Its RI is the highest (RI = 1.52) among all aqueous-based clearing techniques and might improve the final transparency of cleared samples.

### Possible applications of tissue clearing coupled with high through-put imaging

Cleared and fluorescently labeled samples can undergo high through-put imaging. In the field of neuroanatomy, methodologies for quantitative volumetric analyses have been explored by combining tissue clearing, high through-put imaging, and computational tools to handle large volumes of data [30]. Progress in this field allows the collection of large 3D morphometric datasets. These datasets could also be used to generate a 3D reference model, in which anatomical variations among individuals are averaged. This approach would facilitate the quantitative comparison of anatomical characteristics among groups [30, 31].

Various zoological studies could benefit from this approach. For example, developmental biologists could localize cell positions of a species of interest in a 3D space at a given developmental stage [32]. This approach could also be used to evaluate changes to any morphological characteristics caused by exposure to chemicals, genetic mutation, or selection pressure. The library of 3D reference models also has the potential to facilitate the sorting and identification of collected species, and, eventually, our understanding of local fauna [4].

## Conclusions

In this study, we developed a method for the whole-mount clearing of small crustaceans by introducing various pretreatments to an established tissue clearing technique, advanced CUBIC. With species-specific modifications and the development of staining procedures, this method is expected to be a useful tool for morphological investigations in the field of zoology.

## Additional file

Additional file 1: **Figure S1.** Effect of EDTA treatment on the exoskeleton of *A. vulgare* (A), a cheliped of *Philyra* sp. (B), and a leg of *V. analis* (C). (JPG 420 kb)
