# Supplementary figures and images for: Aqueous-based tissue clearing in crustaceans
Source: Zoological Lett. 2018 Jun 6;4:13. doi: 10.1186/s40851-018-0099-6 (PMC5991465; doi:10.1186/s40851-018-0099-6)

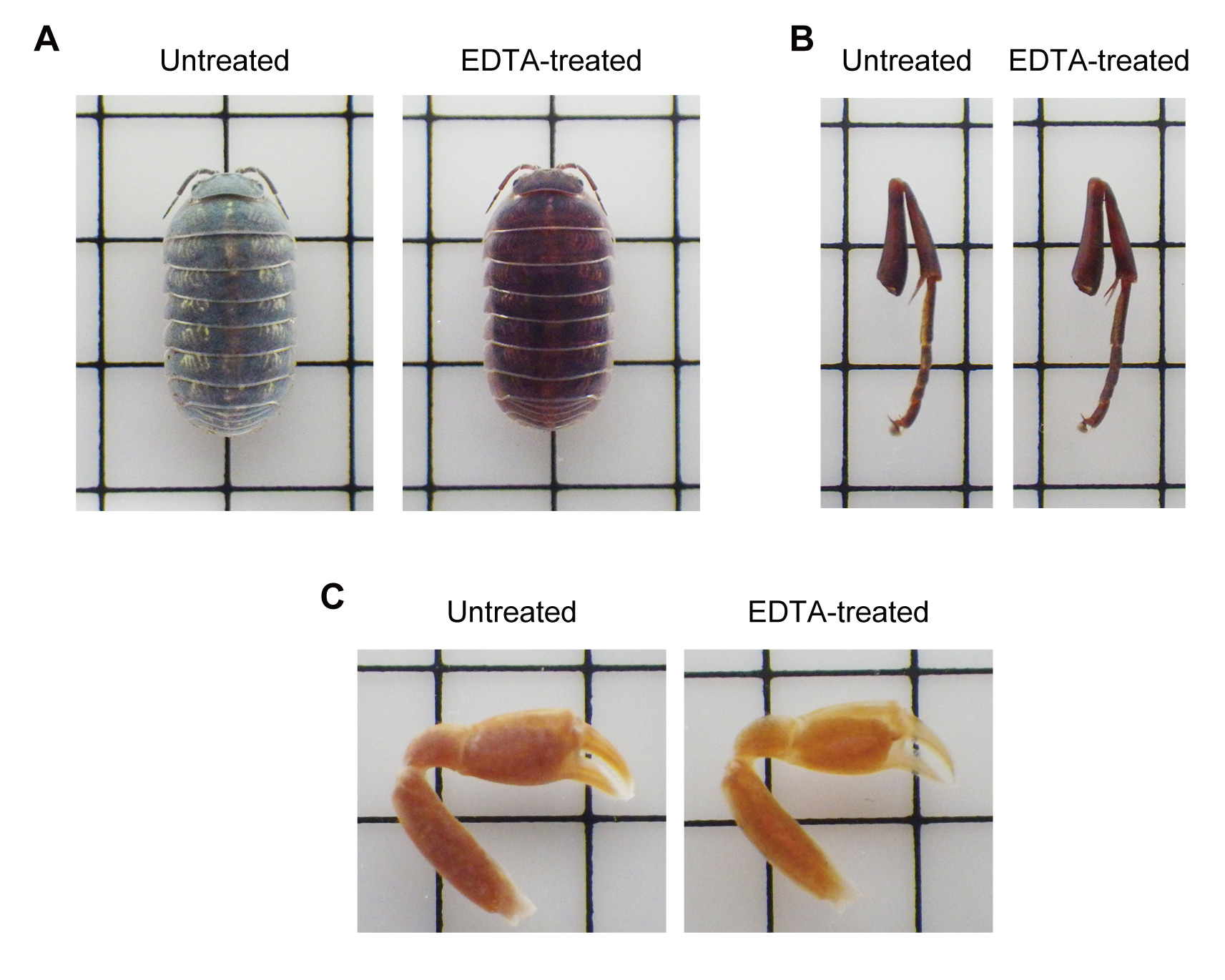

Supplement: Supplementary file 1 — Figure S1. Effect of EDTA treatment on the exoskeleton of A. vulgare (A), a cheliped of Philyra sp. (B), and a leg of V. analis (C). (JPG 420 kb) [file 40851_2018_99_MOESM1_ESM.jpg]
